# Allelic variants of *OsSUB1A* cause differential expression of transcription factor genes in response to submergence in rice

**DOI:** 10.1186/s12284-017-0192-z

**Published:** 2018-01-08

**Authors:** Niharika Sharma, Trang Minh Dang, Namrata Singh, Slobodan Ruzicic, Bernd Mueller-Roeber, Ute Baumann, Sigrid Heuer

**Affiliations:** 1 School of Agriculture, Food and Wine, University of Adelaide, Waite Campus, Plant Genomics Centre, Hartley Grove, Urrbrae, Adelaide, South Australia 5064 Australia; 2 International Rice Research Institute (IRRI), Los Banos, Philippines; 3 Intrexon Corp, California, USA; 4 Section of Plant Breeding and Genetics, School of Integrative Plant Science, Cornell University, Ithaca, NY 14853 USA; 5 University of Potsdam, Potsdam, Germany; 6 Rothamsted Research, Plant Science Department, Hertfordshire, Harpenden, AL5 2JQ UK

**Keywords:** Submergence tolerance, *SUB1A*, Rice, Transcription factors

## Abstract

**Background:**

Flooding during seasonal monsoons affects millions of hectares of rice-cultivated areas across Asia. Submerged rice plants die within a week due to lack of oxygen, light and excessive elongation growth to escape the water. Submergence tolerance was first reported in an *aus*-type rice landrace, FR13A, and the ethylene-responsive transcription factor (TF) gene *SUB1A-1* was identified as the major tolerance gene. Intolerant rice varieties generally lack the *SUB1A* gene but some intermediate tolerant varieties, such as IR64, carry the allelic variant *SUB1A-2*. Differential effects of the two alleles have so far not been addressed. As a first step, we have therefore quantified and compared the expression of nearly 2500 rice TF genes between IR64 and its derived tolerant near isogenic line IR64-Sub1, which carries the *SUB1A-1* allele. Gene expression was studied in internodes, where the main difference in expression between the two alleles was previously shown.

**Results:**

Nineteen and twenty-six TF genes were identified that responded to submergence in IR64 and IR64-Sub1, respectively. Only one gene was found to be submergence-responsive in both, suggesting different regulatory pathways under submergence in the two genotypes. These differentially expressed genes (DEGs) mainly included MYB, NAC, TIFY and Zn-finger TFs, and most genes were downregulated upon submergence. In IR64, but not in IR64-Sub1, *SUB1B* and *SUB1C*, which are also present in the *Sub1* locus, were identified as submergence responsive. Four TFs were not submergence responsive but exhibited constitutive, genotype-specific differential expression. Most of the identified submergence responsive DEGs are associated with regulatory hormonal pathways, i.e. gibberellins (GA), abscisic acid (ABA), and jasmonic acid (JA), apart from ethylene. An *in-silico* promoter analysis of the two genotypes revealed the presence of allele-specific single nucleotide polymorphisms, giving rise to ABRE, DRE/CRT, CARE and Site II *cis*-elements, which can partly explain the observed differential TF gene expression.

**Conclusion:**

This study identified new gene targets with the potential to further enhance submergence tolerance in rice and provides insights into novel aspects of *SUB1A*-mediated tolerance.

**Electronic supplementary material:**

The online version of this article (doi: 10.1186/s12284-017-0192-z) contains supplementary material, which is available to authorized users.

## Background

Rice (*Oryza sativa L.*) is an important food crop globally and the most advanced genetic monocot model amongst the cereal crops (Cantrell and Reeves, 2002). Biotic and abiotic stresses, such as drought and heat, are known to be detrimental to crop production. Rice production is additionally constraint by submergence stress during the rainy season with complete submergence and water stagnation affecting about 20 million hectares of rice fields in the tropics, causing significant yield and economic losses, and food insecurity (Septiningsih et al., 2012). Rice fields can be flooded with several meters of water for weeks and plants die within a few days from lack of oxygen and impaired photosynthesis (Gibbs and Greenway, 2003; Fukao and Bailey-Serres, 2004). Furthermore, in the attempt to escape the water, plants excessively elongate leaves thereby depleting their carbohydrate and energy reserves, and as water recedes, the plants lodge thus impairing any recovery growth (Singh, Singh, and Ram, 2001; Das, Sarkar, and Ismail, 2005; Fukao, Xu, Ronald, and Bailey-Serres, 2006).

Submergence-tolerant rice varieties were identified in the early 1960s and breeding efforts to introgress this trait into rice cultivars have been ongoing since the 1970s. Submergence tolerance was initially considered a complex trait involving many genes located in multiple quantitative trait loci (QTL) (Mackill, Ismail, Singh, Labios, and Paris, 2012). However, advances in molecular marker technologies and optimized phenotyping techniques aided in the identification of the major QTL *Submergence tolerance 1* (*Sub1*) (Xu, Deb, and Mackill, 2004). *Sub1* was identified from the *aus*-type rice landrace FR13A (Flood Resistance 13A) and it provided a breakthrough in the understanding of submergence tolerance mechanisms, enabling marker-assisted breeding of submergence-tolerant rice (Fukao et al., 2006; Xu et al., 2006; Singh, Mackill, and Ismail, 2009; Bailey-Serres et al., 2010; Mackill et al., 2012). Sub1 rice varieties are now widely grown across Asia enhancing plant survival in flooded fields with yield advantages of one ton per hectare or more (Septiningsih et al., 2009; Mackill et al., 2012).

Molecular and comparative sequence analysis of the *Sub1* genomic region in submergence-tolerant and -intolerant rice varieties revealed the presence of a variable cluster of two to three ethylene-responsive TFs (ERFs) genes, namely *SUB1A*, *SUB1B* and *SUB1C* (Xu et al., 2006). In submergence-intolerant and moderately tolerant rice genotypes, the *SUB1A-1* gene is either absent, due to an inversion-deletion, or present as an allelic variant, *SUB1A-2* (Singh et al., 2010) (Additional file 1: Figure S1a).

Transgenic approaches identified *SUB1A-1* as the major tolerance gene, since its constitutive expression conferred submergence tolerance to an intolerant rice variety (M202), which naturally lacks the *SUB1A* gene (Xu et al., 2006). Under complete submergence, the main phenotypic effect observed in *SUB1A-1* overexpression and *Sub1* near isogenic lines (NILs), is a significantly reduced elongation growth (Additional file 1: Figure S1b). The Sub1 plants assume a “quiescence” status which prevents excessive elongation growth thereby preserving carbohydrate reserves and preventing an energy crisis, as well as lodging once the water recedes (Fukao et al., 2006; Xu et al., 2006; Voesenek and Bailey-Serres, 2009).

In contrast to the *SUB1A*-mediated tolerance, deepwater rice responds to submergence with rapid GA-induced elongation growth, so that plants reach the water surface for access to light and oxygen (Fukao et al., 2006; Xu et al., 2006; Bailey-Serres and Voesenek, 2008; Singh et al., 2010). As is the case for *SUB1A-1,* rapid elongation growth in deepwater rice is also ethylene induced but regulated by different ERFs, namely *SNORKEL1* (*SK1*) and *SNORKEL2 (SK2*) (Hattori et al., 2009; Nagai, Hattori, and Ashikari, 2010).

At the molecular level, *SUB1A-1* functions by preventing ethylene-induced, GA-mediated elongation growth via enhancing the level of the GA-inhibitors *SLENDER RICE 1* (*SLR1*) and *SLENDER RICE LIKE 1* (*SLRL1*) (Fukao and Bailey-Serres, 2008). Further to this, presence of *SUB1A-1* is associated with the negative regulation of genes involved in carbohydrate catabolism and cell elongation (via expansins) and positive regulation of genes involved in ethanolic fermentation, such as alcohol dehydrogenase (Fukao et al., 2006; Bailey-Serres and Voesenek, 2010). Recently some studies have revealed that *SUB1A-1* is phosphorylated by *MITOGEN-ACTIVATED PROTEIN KINASE3* (*MAPK3*) and in turn physically interacts with MAPK3 and binds to its promoter (Singh and Sinha, 2016). Overall, the suppressed growth and metabolic adjustments during flooding confers a higher recovery rate to Sub1 plants compared to non-Sub1 plants as illustrated in the aerial photo of the IRRI demonstration plot (Additional file 1: Figure S1b).

The aforementioned molecular and physiological studies were conducted by comparing Sub1 NILs and *SUB1A-1* overexpressing plants with their corresponding wild-type varieties (M202 and Liaogeng, respectively), which have the *SUB1B* and *SUB1C* genes but naturally lack the *SUB1A* gene (Fukao et al., 2006; Xu et al., 2006). However, an allelic variant of the *SUB1A* gene exists, *SUB1A-2*, which is present in a range of rice varieties (Fukao et al., 2006; Singh et al., 2010). Phenotyping of these *SUB1A-2* varieties revealed a variable level of submergence tolerance ranging from about 4% to 40% plant survival after two weeks of submergence. *SUB1A* expression analysis subsequently showed that both, *SUB1A-1* and *SUB1A-2* are submergence-inducible and expressed at similar levels in leaves, stems and developing panicles. However, gene expression in internodes associated well with submergence tolerance, i.e., a higher level of *SUB1A* expression in internodes under submergence was observed in varieties with the *Sub1A-1* allele compared to varieties with the *SUB1A-2* allele (Singh et al., 2010) (Additional file 1: Figure S1c).

To further address differences between *SUB1A-1* and *SUB1A-2* allele effects, we analysed internode samples from IR64 (*SUB1A-2*) and the IR64-derived NIL IR64-Sub1, which carries the tolerant *Sub1* locus including the *SUB1A-1* allele (Singh et al., 2010). Quantitative real-time PCR (qPCR) targeting nearly 2500 rice TF genes based on the Nipponbare reference genome (Caldana, Scheible, Mueller-Roeber, and Ruzicic, 2007) was conducted with internode samples from plants submerged for 30 h and corresponding non-submerged control plants to (a) identify TFs that are differentially regulated upon submergence in IR64 and IR64-Sub1, (b) compare TFs in both genotypes to provide insights in the SUB1A regulatory pathway and (c) analyse the promoter region of S*UB1A-1* and *SUB1A-2* to identify single nucleotide polomorphisms (SNPs) that create putative *cis*-elements and could be binding sites for upstream TFs.

## Methods

### Plant material and submergence treatment

Seeds of the rice variety IR64 (accession number 66970) and the IR64-Sub1 NIL (accession number IR8419422–139) were provided by the International Rice Germplasm Collection (IRGC) at IRRI, Philippines. Seeds were incubated at 55 °C for 5 days to break dormancy before pre-germination in petri dishes at 37 °C in an incubator. Three-day-old seedlings were transplanted into pots filled with soil substituted with 3 g ammonium sulphate. Four pots with two plants for each accession were grown for 75 days until booting/heading stage, i.e., the onset of internode elongation. At that stage, plants were completely submerged in a concrete tank filled with about 2 m of tap water for 30 h (Singh et al., 2010). Non-submerged control plants were kept under natural conditions in an IRRI screenhouse and sampled in parallel. From each plant, multiple internodes from three tillers were sampled from a total of four submerged and two control plants per genotype. Samples were immediately frozen in liquid nitrogen and stored at −80°C.

### RNA isolation and cDNA preparation

Total RNA was isolated from the internode tissues of the control and submerged plants using TRIzol® (Invitrogen) according to the manufacturer’s instructions. The quantity and quality of RNA samples were assessed using a Nanodrop ND-100 (Thermo Scientific) and by agarose gel electrophoresis. DNA contamination was removed by treating RNA samples with RNase-free DNase I according to the protocol provided (Promega). cDNA was synthesized from 5 μg of total RNA using Superscript™ III reverse transcriptase (Invitrogen, Germany) as per the manufacturer’s protocol.

### Data source for transcription factor sequences and primers

A qRT-PCR platform was used for expression profiling of the rice TF genes (Caldana et al., 2007). The initial source dataset used for the annotation and primer design was version 2.0 of the Rice Genome Annotation Project (RGAP) (http://rice.plantbiology.msu.edu/) and encompassed a total of 2508 (2487 unique) TF genes. TF sequences were extracted from version 1 of the Plant Transcription Factor Database (Riano-Pachon, Ruzicic, Dreyer, and Mueller-Roeber, 2007; Perez-Rodriguez et al., 2010). The TF genes were initially synchronized with version 5.0 of the RGAP Pseudomolecule and genome annotations resulting in 2221 unique genes with appropriate annotation in version 5.0. Twenty of these were represented in two distinct positions in the qPCR platform as internal controls. Based on version 5.0 of the RGAP Pseudomolecules, 266 unique genes out of 2487 missed annotations (Additional file 2: Table S1). The TF genes were further revised based on the most recent RGAP version 7.0 annotation and details are given in Additional file 2: Table S2. For the genes with missing annotations in Release 7, BLAST searches were performed taking the sequences of the PCR amplicons as queries against the RGAP database.

### Transcription factor profiling

The qPCR platform used for this study had the primer pairs specific to the TF genes described above and were distributed on a total of seven 396 well-plates (Caldana et al., 2007). On each plate, twelve wells were reserved for three reference genes (RGs; represented in quadruplicate) and four wells were for negative water control. For the current study, the RGs 9631.m04973, 9629.m05807 and 9633.m03388 were used (Additional file 2: Table S3 and S4). Of these, 9633.m03388 (LOC_Os05g36290, actin) was selected as the best suitable RG for all subsequent calculations and normalizations. There were three technical replicates for each of the four samples (IR64_C_, IR64_S_, IR64-Sub1_C_ and IR64-Sub1_S_, where C denoted control and S submergence treated plants). Real-time PCR analysis was based on SYBR Green (Applied Biosystems, Germany) and was conducted in a total volume of 5 μl (2.5 μl of SYBR Green master mix, 0.5 μl of cDNA (1.25 ng/μl) and 200 nM forward and reverse primers). PCR amplifications were carried out as described in (Caldana et al., 2007) in an ABI PRISM 7900 HT sequence detection system (Applied Biosystems, Germany).

### Data processing and statistical analysis

Based on the obtained amplification curves, the Ct values (fractional cycle number at threshold), the value of PCR efficiency and the corresponding coefficient of determination (R^2^) were calculated using LinRegPCR software (Ruijter et al., 2009) for all four samples (IR64_C_, IR64_S_, IR64-Sub1_C_ and IR64-Sub1_S_). Ct values for all reactions were log_2_ transformed (Additional file 2: Table S3 and S4). The average Ct values for RGs (Ct_RG_) were calculated from Ct values of quadruplicates for each 396 well-plate separately for control and submerged conditions. The RG selected for all subsequent calculations was actin (LOC_Os05g36290). Based on the average Ct_RG_, the ΔCt for each selected gene (SG) was calculated as ΔCt = Ct_SG_ - Ct_RG_. All reactions with R^2^ < 0.995, reflecting low-quality amplifications and those designated as “undetermined” (did not deliver a Ct < 40) were excluded from further analyses. The obtained sets of ΔCt values for control and submerged conditions for each gene model (in three replicates) were examined for significant differential expression using LIMMA’s moderated t-test (Smyth, 2005). LIMMA computes moderated t-statistics and log-odds of differential expression by empirical Bayes shrinkage of the standard errors towards a common value. False Discovery Rate (FDR) corrections of *p*-values were carried out using Benjamini and Hochberg method (Benjamini and Hochberg, 1995). A gene was considered differentially expressed if the moderated t-test resulted in a corrected p-value <0.01. Hence, the list of differentially expressed TF genes and corresponding log_2_FC (fold change) values under submergence stress were generated from both, the IR64 and the IR64-Sub1 submerged plants compared to the respective control plants. In addition, we examined the data for genotype-specific expression differences. For this, we used the contrast matrix referred from the LIMMA manual (https://www.bioconductor.org/packages/devel/bioc/vignettes/limma/inst/doc/ usersguide.pdf) as follows:

Contrast.matrix <− makeContrasts (SubmergenceIR64 = IR64_S_-IR64_C_, SubmergenceIR64Sub1 = IR64Sub1_S_ - IR64Sub1_C_, Genotype = (IR64Sub1_S_ + IR64Sub1_C_) - (IR64_S_ + IR64_C_) levels = design).

### Promoter analysis

The promoter sequences of the *SUB1A-1 and SUB1A-2* alleles (Additional file 2: Table S5) were aligned using Clustal Omega (http://www.ebi.ac.uk/Tools/msa/clustalo/) and analysed using PlantPAN 2.0 (Chow et al., 2016) as well as manually. A 2 kb upstream DNA region of the DEGs was extracted from RAP-DB (http://rapdb.dna.affrc.go.jp/index.html) and analysed by PlantPAN (http://plantpan2.itps.ncku.edu.tw/) to identify putative *cis-*regulatory elements.

## Results

In order to investigate the difference between submergence tolerance as mediated by the strong allele *SUB1A-1* (present in IR64-Sub1) and the weak allele *SUB1A-2* (present in IR64), expression of 2487 rice TF genes was quantified by qPCR in internodes of submerged and non-submerged control plants. Internodes were chosen for this study since differences in the expression of the *SUB1A-1* and *SUB1A-2* alleles were most pronounced in this tissue (Singh et al., 2010).

### Update of TF gene annotations

The 2487 TF genes given in Additional file 2: Table S1 were synchronised to the latest version 7.0 of the RGAP Pseudomolecules and annotation of the rice genome (Kawahara et al., 2013). For 72 of the 266 genes with missing annotations, we could obtain annotations from version 7.0 (Additional file 2: Table S2) and one gene was marked as obsolete. Therefore, only 193 genes remained as not being annotated in version 7.0. Nine genes previously annotated in version 5 miss annotations in version 7, eight of these are now considered obsolete and one is without annotation in versions 6.1 and 7.0 of RGAP annotations. Hence, a total of 2284 TF gene models were updated with version 7.0 annotations (Table 1).

**Table 1:** Summary of the total number of genes with and without annotations in the RGAP pseudomolecules version 7.0

Number of TF Genes with and without annotations in version 7.0 of RGAP annotations					
Version 5.0 annotation from RGAP					
Genes	All	Missing annotations		With annotations	
Total	2508	267		2241	
Unique	2487	266		2221	
Version 7.0 annotation from RGAP					
		Obsolete	With annotations	Obsolete	Without annotations
Unique		1	72	8	1
		193 missing annotations		2212 annotated	
Total				2284 annotated genes	

### Differential gene expression analysis in IR64 and IR64-Sub1

**Fig. 1:**
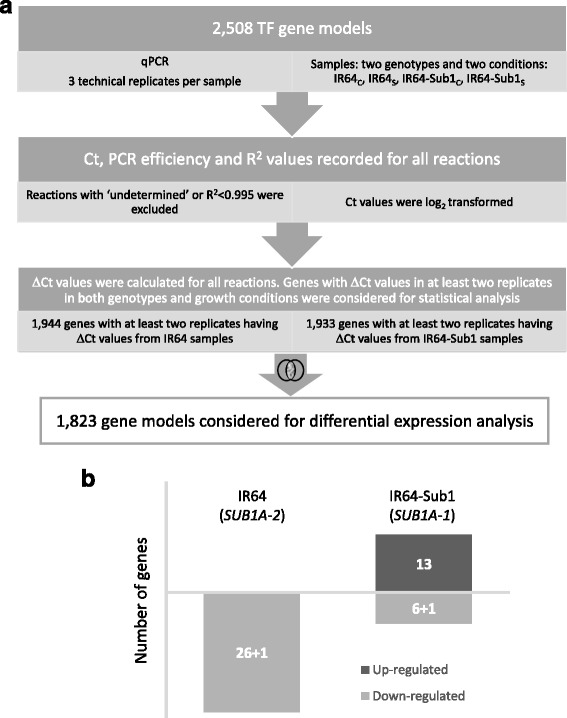
Identification of differentially expressed transcription factor genes. **a** Overview of the data processing and selection of genes as input for the statistical analysis of differential gene expression using LIMMA. **b** Differentially expressed transcription factor genes under submergence stress in IR64 and IR64-Sub1. +1 indicates the common TF gene (MYB), which is submergence-responsive in both genotypes

The expression patterns of the TF genes in four samples (IR64_C_, IR64_S_, IR64-Sub1_C_ and IR64-Sub1_S_) were analysed by qPCR. Statistical comparisons for the identification of DEGs were performed only for those genes which possessed at least two calculated ΔCt values in both genotypes and both growth conditions (control and submerged). For performing differential expression analysis an intersect of 1823 TF genes, 1944 genes of the IR64 dataset and 1933 of the IR64-Sub1 dataset, were taken as input for LIMMA’s moderated t-test comparisons (Fig. 1a). The resulting *p*-values, FDR corrected p-values and log_2_FC values for all gene models are provided in Additional file 2: Table S6.

### Genotype-specific expression differences of TFs

**Fig. 2:**
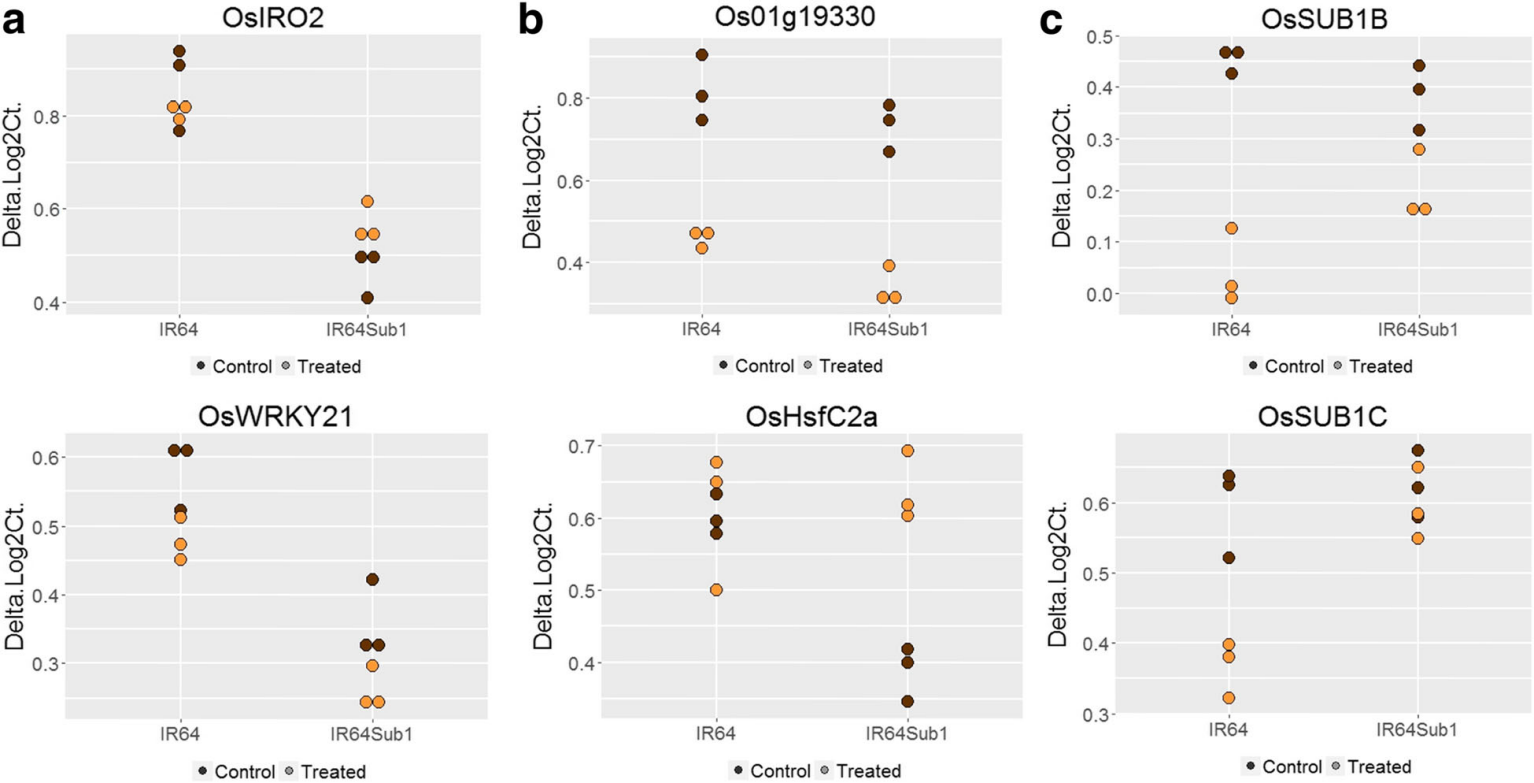
Differentially expressed transcription factor genes in IR64 and IR64-Sub1. Representative dot plots are shown illustrating three different categories of DEGs: **a** Non-submergence-responsive genes with genotype-dependent differential expression, (**b**). submergence-responsive genes in IR64-Sub1, (c). and submergence-responsive genes in IR64

The genotypic comparison revealed a total of four TF genes that were not submergence responsive but depicted genotype-dependent expression differences between IR64 and IR64-Sub1 (Table 2). These genes code for a basic helix-loop-helix (bHLH) factor *OsIRO2* (LOC_Os01g72370)*, OsWRKY21* (LOC_Os01g60640) and two TIFY type TFs (*OsTIFY11e/OsZIM18/OsJAZ13* (LOC_Os10g25230)*; OsTIFY11d/OsJAZ12* (LOC_Os10g25290)). All four genes had overall lower expression levels in IR64-Sub1 compared to IR64 (Fig. 2a).

**Table 2:** Details of the differentially expressed TF genes and their corresponding fold change value (log2FC) under submergence in IR64 and IR64-Sub1

	Gene #	Locus ID ver 7.0	Gene Description/TF Family	Gene name	og2FC
Genotype specific differences	1	LOC_Os01g72370	Helix-loop-helix DNA-binding domain containing protein; bHLH	OsIRO2/OsbHLH056	−0.64
	2	LOC_Os10g25230	ZIM domain containing protein; Tify	OsTIFY11e/OsZIM18/OsJAZ13	−0.50
	3	LOC_Os10g25290	ZIM domaincontaining protein; Tify	OsTIFY11d/OsJAZ12	−0.36
	4	LOC_Os01g60640	WRKY21; WRKY	OsWRKY21	−0.44
Submergence responsive in IR64	1	LOC_Os07g22730	AP2 domain containing protein; AP2-EREBP	OsERF136/AP2/EREBP#106	−0.19
	2	LOC_Os05g27930	AP2 domain containing protein; AP2-EREBP	OsERF042/AP2/EREBP#048/OsDREB2b	−0.28
	3	LOC_Os04g47059	bHLH	OsbHLH16/OSB2	0.26
	4	LOC_Os01g36220	bZIP transcription factor domain containing protein; bZIP	OsbZIP4	0.31
	5	LOC_Os02g35770	Homeobox associated leucine zipper; HB	OsHox7	0.18
	6	LOC_Os02g13800	HSF-type DNA binding domain containing protein; HSF	OsHsfC2a/OsHsf-05	0.25
	7	LOC_Os02g40530	MYB family transcription factor; MYB	MYB/OsMPS	−0.28
	8	LOC_Os07g37210	MYB family transcription factor; MYB	MYB/OsMyb7	0.17
	9	LOC_Os11g47460	MYB family transcription factor; MYB	MYB	0.16
	10	LOC_Os06g51260	MYB family transcription factor; MYB-related	MYB/ OsLHY-like_chr.6	0.36
	11	LOC_Os01g47370	MYB family transcription factor; MYB-related	MYB	0.20
	12	LOC_Os08g06110	MYB family transcription factor; MYB-related	MYB/OsCCA1/OsLHY/LHY-like_chr.8	0.33
	13	LOC_Os07g26150	MYB family transcription factor; MYB-related	MYB/Hsp40	0.18
	14	LOC_Os04g41560	B-box zinc finger family protein; Orphans	OsBBX11/OsSTO	0.21
	15	LOC_Os08g08120	B-box zinc finger family protein; Orphans	OsBBX24	0.21
	16	LOC_Os09g25060	WRKY76; WRKY	OsWRKY76	−0.31
	17	LOC_Os03g08310	ZIM domain containing protein; Tify	OsTIFY11a/OsJAZ9	−0.31
	18	LOC_Os02g35329	RING-H2 finger protein ATL3F	ATL3F/OsELF5	−0.28
	19	LOC_Os07g43740	Zinc finger, C3HC4 type domain containing protein	Zn finger	0.20
Common gene	1	LOC_Os01g19330	MYB family transcription factor; MYB	MYB	−0.394
					−0.36
Submergence responsive in IR64	1	LOC_Os08g06120	B3 DNA binding domain containing protein; ABI3VP1	Similar to NGA1 (NGATHA1)	−0.26
	2	LOC_Os09g11480	AP2 domain containing protein; AP2-EREBP	OsSUB1B/OsERF063/AP2/EREBP#166	−0.41
	3	LOC_Os09g11460	AP2 domain containing protein; AP2-EREBP	OsSUB1C/ OsERF73/AP2/EREBP#122	−0.23
	4	LOC_Os07g47790	AP2 domain containing protein; AP2-EREBP	OsERF067/AP2/EREBP#076	−0.22
	5	LOC_Os03g22170	AP2 domain containing protein; AP2-EREBP	OsERF066/AP2/EREBP#030	−0.31
	6	LOC_Os01g54890	Ethylene-responsive transcription factor 2; AP2-EREBP	OsERF922/AP2/EREBP#078	−0.21
	7	LOC_Os01g64020	Transcription factor; bZIP	OsLG2/OsbZIP11	−0.16
	8	LOC_Os02g49230	CCT/B-box zinc finger protein; C2C2-CO-like	OsBBX7	−0.17 −0.18 −0.20
	9	LOC_Os01g74410	MYB family transcription factor; MYB	OsMYB48–1	−0.23 −0.32
	10	LOC_Os03g20090	MYB family transcription factor; MYB	OsMYB2	−0.18
	11	LOC_Os08g43550	MYB family transcription factor; MYB	OsMYB7	−0.24
	12	LOC_Os03g04900	MYB family transcription factor; MYB	MYB	−0.20
	13	LOC_Os11g05614	No apical meristem protein; NAC	OsONAC7/ONAC17/ONAC30/OsNAC111	−0.26
	14	LOC_Os07g37920	No apical meristem protein; NAC	OsSNAC/ONAC10/OsSTA199	−0.18
	15	LOC_Os03g04070	No apical meristem protein; NAC	OsANAC34/ONAC22	−0.21
	16	LOC_Os01g66120	No apical meristem protein; NAC	OsNAC6/SNAC2/NAC48	−0.26
	17	LOC_Os01g10580	B-box zinc finger family protein; Orphans	OsBBX1/OsDBB3c	−0.22
	18	LOC_Os12g10660	B-box zinc finger family protein; Orphans	OsBBX30	−0.21
	19	LOC_Os02g05470	CCT motif family protein; Orphans	OsCCT03/OsCMF3	−0.43
	20	LOC_Os07g07690	PHD-finger domain containing protein; PHD	PHD	−0.43
	21	LOC_Os05g49620	WRKY19; WRKY	OsWRKY19	−0.25
	22	LOC_Os09g25070	WRKY62; WRKY	OsWRKY62	−0.33
	23	LOC_Os02g08440	WRKY71; WRKY	WRKY71/OsEXB1	−0.25
	24	LOC_Os09g30400	WRKY90; WRKY	OsWRKY90	−0.18
	25	LOC_Os06g03580	Zinc RING finger protein	OsBBI1	−0.20
	26	LOC_Os01g11460	Zinc finger, C3HC4 type domain containing protein	Zn finger ATL2K	−0.20

**Fig. 3:**
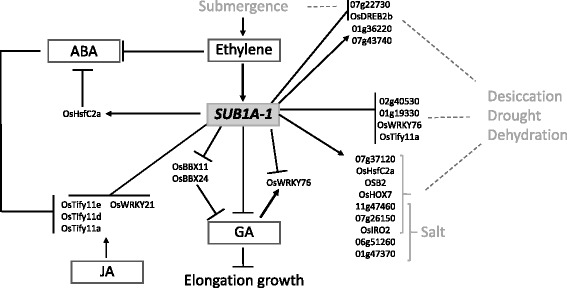
Differentially expressed transcription factor genes in IR64-Sub1. An overview of the transcription factor genes and their related pathways is provided for all genes that are specifically submergence-responsive in IR64-Sub1 which carries the *SUB1A-1* allele. The figure is based on a literature review, see text for details

**Fig. 4:**
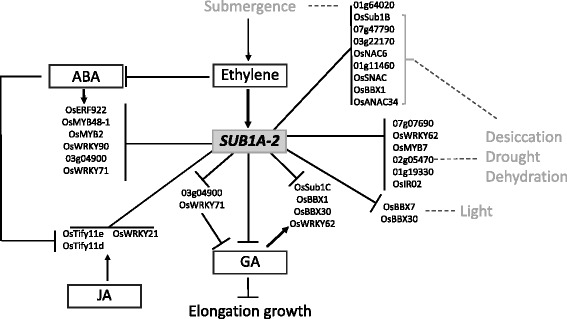
Differentially expressed transcription factor genes in IR64. An overview of the transcription factor genes and their related pathways is provided for all genes that are specifically submergence-responsive in IR64 which carries the *SUB1A-2* allele. The figure is based on a literature review, see text for details

Studies have shown these four TF genes to be regulated (upregulated - ↑ or downregulated- ↓) under the following conditions: iron (Fe) starvation (*OsIRO2* ↑); desiccation/drought (*OsIRO2* ↓*, TIFY11d & 11e* ↑); salinity (*OsIRO2* ↓*, TIFY11d & 11e* ↑); salicylic acid (SA) (*OsWRKY21* ↑); wounding (*TIFY11d & 11e* ↑); pathogens (*TIFY11d & 11e* ↑); cold (*TIFY11d & 11e* ↑); JA (*TIFY11d & 11e* ↑), GA (*TIFY11e* ↓) and ABA (*TIFY11d & 11e* ↓) (Ogo et al., 2006; Wang et al., 2013; Ramamoorthy, Jiang, Kumar, Venkatesh, and Ramachandran, 2008; Ye, Du, Tang, Li, and Xiong, 2009; Ranjan et al., 2015; Yang, Chen, Jen, Liu, and Chang, 2015; Shankar, Bhattacharjee, and Jain, 2016; Xiang et al., 2017). Under submergence, *OsTIFY11e* was reported to be downregulated in M202-Sub1 compared to M202 but upregulated under drought (Fukao, Yeung, and Bailey-Serres, 2011). Information on these genes is summarized in Figs. 3 and 4 and details are given in Additional file 2: Table S7.

### Submergence-responsive TFs in IR64-Sub1

In IR64-Sub1, 19 TF genes were found to be submergence-responsive, of which thirteen genes were upregulated and six genes were downregulated (Fig. 1b, Table 2). The group of upregulated genes include: 6 MYB factors, 3 Zn finger TFs, 1 Heat Shock Factor (HSF), 1 bHLH, 1 bZIP, and 1 HOX TFs. And the seven (6 + 1) downregulated genes include: 2 AP2-type TFs, 2 MYBs, a WRKY, a TIFY and a RING-H2 finger gene. One of the seven downregulated genes, the MYB factor LOC_01g19330, was downregulated in response to 30 h of submergence in both IR64 and IR64-Sub1 genotypes and is placed in the category of a common gene (Fig. 2, Table 2). Studies of these TFs in relation to abiotic stress and hormonal response are detailed below and an overview of the data is provided in Fig. 3.

#### Upregulated genes

MYB TFs have been reported to play various biological functions in plants. With respect to abiotic stresses and hormonal pathways the following have been documented in rice: salinity stress (LOC_Os06g51260 ↓, LOC_Os01g47370 ↓, LOC_Os07g26150 ↓); GA (MYB_LOC_Os11g47460 ↓, LOC_Os01g47370 ↓), desiccation/drought showed both up and downregulated depending on variety analysed (MYB_LOC_Os07g37210 ↑↓, MYB_LOC_Os11g47460 ↑↓, MYB_LOC_Os07g26150 ↓); and seed dormancy regulated via ABA-GA antagonism (MYB_LOC_ Os08g06110 ↑) (Baxter et al., 2007; Gao et al., 2009; Katiyar et al., 2012; Boyden et al., 2012; Wu et al., 2016; Park et al., 2015; Shankar et al., 2016; Xiang et al., 2017).

The Zn finger TFs included two B-Box domain genes, namely *OsBBX11* (LOC_Os04g41560) and *OsBBX24* (LOC_Os08g08120), and one gene with a Zn-finger binding domain (LOC_Os07g43740). *OsBBX11* has been reported to have two BBX domains, whereas *OsBBX24* has one BBX domain. These two genes are responsive to various hormones; auxin (*OsBBX11* ↓); GA (*OsBBX11* ↓, *OsBBX24* ↓); cytokinin (*OsBBX11* ↓); kinetin (*OsBBX11* ↓, *OsBBX24* ↓↓) and naphthalene acetic acid (NAA) (*OsBBX24* ↓). *OsBBX11* was also seen to be upregulated by light and downregulated by desiccation (Huang, Zhao, Weng, Wang, and Xie, 2012; Shankar et al., 2016). LOC_Os07g43740 has been document to be upregulated under several abiotic stresses, i.e. anoxia, drought, desiccation, salt, heat and chilling (Ham, Moon, Hwang, and Jang, 2013; Shankar et al., 2016).

The heat shock factor (HSF) gene, *OsHsfC2a* (LOC_Os02g13800), which was upregulated upon submergence in IR64-Sub1, has also been reported to have increased expression under other abiotic stresses, i.e. heat, cold, oxidative stress (Mittal et al., 2009; Mittal et al., 2012) as well as drought and desiccation (Chauhan, Khurana, Agarwal, and Khurana, 2011; Shankar et al., 2016). This TF also showed upregulation in response to brassinosteroids (BR) and SA but the expression decreased in response to ABA (Chauhan et al., 2011; Shankar et al., 2016). Promoter analysis of *OsHsfC2a* revealed presence of light, ABA and methyl-jasmonate (MeJA) responsive *cis*-elements (Wang, Zhang, and Shou, 2009).

The bHLH *OsbHLH016*/*OSB2* (LOC_Os04g47059) and the HOX *OsHOX7* (LOC_Os02g35770) TF genes show upregulation under desiccation stress (Shankar et al., 2016), whereas no information on the bZIP factor (LOC_Os01g36220) is available.

Only two of the above-mentioned genes have previously been reported to be upregulated under submergence and dehydration in IR64-Sub1, namely the bZIP gene (LOC_Os01g36220) and the Zn-finger gene (LOC_Os07g43740) (Fukao et al., 2011). The latter is also upregulated under anoxia, heat and chilling (Ham et al., 2013; Shankar et al., 2016).

#### Downregulated genes

In this category, two AP2-type TFs (LOC_Os07g22730, LOC_Os05g27930) have previously been reported as submergence-responsive (Jung et al., 2010). LOC_Os07g22730 belongs to the AP2 group IIIb and was slightly upregulated in M202-Sub1 after 6d of submergence but downregulated under anoxia. This gene is also upregulated under desiccation and salt stress (Shankar et al., 2016) and known to be highly expressed in roots and embryos (Rashid, He, Yang, Hussain, and Yan, 2012). The other AP2 factor (LOC_Os05g27930) codes for *OsDREB2b*, which was reported to be slightly downregulated under submergence in both, M202 and M202-Sub1 (Jung et al., 2010). *OsDREB2b* is a transcriptional activator and is upregulated in the nodes of drought-stressed plants (Todaka et al., 2012). It is also responsive to high and low-temperature stress and over-expressing lines exhibit increased plant survival under drought (Matsukura et al., 2010).

The two downregulated MYB TF genes (LOC_Os02g40530, LOC_01g19330) encode putative calmodulin-binding proteins and are known to play important roles in signal transduction regulating plant development and adaptation responses to different stress conditions (Chantarachot, Buaboocha, Gu, and Chadchawan, 2012). Both genes were reported to be upregulated under drought, desiccation and salt stress whereas downregulated when treated with GA (Chantarachot et al., 2012; Katiyar et al., 2012; Shankar et al., 2016; Xiang et al., 2017).

The other downregulated genes in this category code for *OsWRKY76* (LOC_Os09g25060), *OsTIFY11a*/*OsJAZ9* (LOC_Os03g08310) and a RING-H2 finger gene (LOC_Os02g35329). *OsWRKY76* is constitutively expressed in vegetative and reproductive tissues and upregulated under desiccation, salinity, auxin, GA and MeJA treatments (Ramamoorthy et al., 2008; Shankar et al., 2016). In contrast, *OsTIFY11a*/JAZ9 is specifically expressed in young panicles (Ye et al., 2009). *OsTIFY11a*/JAZ9 is widely stress-responsive and induced by JA, wounding, drought, desiccation, salt and cold stress but downregulated by ABA treatment (Shankar et al., 2016). It was shown that overexpression of *TIFY11a*/*JAZ9* enhances shoot growth in rice under salt and mannitol treatment (Ye et al., 2009). OsTIFY11a/JAZ9 interacts with OsCOI1 suggesting its involvement in JA signalling as a transcriptional regulator for salt stress tolerance (Wu, Ye, Yao, Zhang, and Xiong, 2015). No information was found for the RING-H2 finger gene.

### Submergence-responsive TFs in IR64

There were 26 submergence-responsive TF genes specific to IR64, excluding the above-mentioned MYB factor LOC_01g19330 common to both genotypes. The genes encode: 5 AP2 domain TFs, 4 NAC, 4 WRKY, 4 MYB, 1 B3 DNA binding protein and a number of different Zn-finger proteins. All of these genes were downregulated under submergence (Fig. 1b; Table 2) and an overview of the data is provided in Fig. 4. Documented studies of these TF genes are summarised below based on their response to submergence, hormone signalling pathways and other abiotic and biotic stresses.

The most responsive gene in IR64 was LOC_Os07g07690, an uncharacterized gene with a putative Jas-domain (pham16135), plant homeodomain (PHD) and Zn-finger domain (cd15539, pfam00628, smart00249). The second most downregulated gene, interestingly, was *SUB1B* (LOC_Os09g11480), one of the three ERF factors within the *Sub1* QTL (Xu et al., 2006) (Table 2, Fig. 2 and Additional file 1: Figure S1a). *SUB1B* is reportedly not responsive to GA or ethylene (Fukao et al., 2006; Xu et al., 2006). The other AP2 TF genes include *SUB1C* (LOC_Os09g11460) and three additional ERFs (LOC_Os03g22170, LOC_Os07g47790 and *OsERF922*: LOC_Os01g54890). The *SUB1C* gene was reported to be induced by GA and ethylene and upregulated under submergence in the intolerant *japonica* variety M202, which naturally lacks the *SUB1A* gene (Fukao et al., 2006). The same study also suggested that *SUB1A-1* negatively regulates *SUB1C* expression. Our results are in agreement with that finding and suggest that *SUB1A-2* also negatively regulates *SUB1C* under submergence (Fig. 2). However, downregulation of *SUB1C* was not observed in IR64-Sub1 suggesting an alternate regulatory pathway. One of the recent studies revealed that desiccation stress could also downregulate *SUB1C* and *SUB1B* in IR64 plants (Shankar et al., 2016). The three other AP2/ERF genes have also been reported from studies on submergence tolerance. *OsERF067* (LOC_Os07g47790) was shown to be upregulated in M202 under submergence and in IR64 under dehydration. This gene was seen to be upregulated by *SUB1A* during submergence and dehydration in M202-Sub1 and IR64-Sub1 suggesting a downstream position in the *SUB1A* pathway (Fukao et al., 2011). Contrastingly, another study reported downregulation of *OsERF067* in M202 under submergence but upregulation in M202-Sub1 (Jung et al., 2010). The same study showed a similar expression pattern for *OsERF066* (LOC_Os03g22170). Both of these AP2 factors are downregulated under GA treatment (Xiang et al., 2017), involved in cytokinin response (Lara et al., 2004) and are positively responsive to anaerobic germination in Nipponbare (Magneschi and Perata, 2009). The gene *OsERF922* was shown to be upregulated in M202-Sub1 after 6 days of submergence but downregulated in M202 after 1 day of submergence (Jung et al., 2010). *OsERF922* is strongly induced by ABA, salt treatment, and biotic stress. A study on the crosstalk between abiotic and abiotic stress signalling through modulation of ABA levels further suggests that *OsERF922* functions as a transcriptional activator (Liu, Chen, Liu, Ye, and Guo, 2012).

Of the 4 NAC genes downregulated in IR64, two have been reported to respond to various biotic and abiotic stresses, including submergence. *OsANAC34* (LOC_Os03g04070) is upregulated under submergence, desiccation and by rice stripe virus infection but downregulated under GA (Nuruzzaman et al., 2010; Shankar et al., 2016; Xiang et al., 2017). The *OsNAC6/SNAC2* (LOC_Os01g66120) gene contains ABRE and CBE elements in its promoter and is upregulated under drought, cold and submergence stress (Nuruzzaman et al., 2010). These two genes are responsive in Nipponbare, suggesting they are not under the control of *SUB1A,* which is absent in this rice variety. *OsNAC6/SNAC2* was also reported to be upregulated under desiccation and salinity stress in a drought-tolerant rice (N22) mutant (Shankar et al., 2016).

The C3HC4 Zn finger (LOC_Os01g11460) and B-Box Zn finger *OsBBX1* (LOC_Os01g10580) TF genes were upregulated under submergence in the intolerant variety M202, the former also in M202-Sub1, and in IR64 upon dehydration (Fukao et al., 2011). *OsBBX1* is highly expressed in seeds, roots and the endosperm, and is induced by auxin, GA and cytokinin (Huang et al., 2012). The bZIP TF factor LOC_Os01g64020 was found to be downregulated under submergence in IR64 plants in our study and was reported to be nitrogen-responsive in studies in rice and Arabidopsis (Obertello, Shrivastava, Katari, and Coruzzi, 2015).

The induction of ethylene in response to submergence is known to contribute to a decline in ABA and an increase in GA thereby regulating elongation growth (see above). ABA in-turn is involved in the regulation of the crosstalk between JA and SA signalling pathways (Liu et al., 2012). A complex interplay and fine-tuning of hormonal responses is therefore likely to determine the level of submergence tolerance.

Several studies have shown that four of the IR64-downregulated TFs are upregulated (↑) or downregulated (↓) in response to ABA: *OsMYB48–1* (LOC_Os01g74410) ↑; R2R3-type *OsMYB2* gene (LOC_Os03g20090) ↑; *OsWRKY90* (LOC_Os09g30400) ↑ and *OsERF066* (LOC_Os03g22170) ↑. These genes were also reported to be responsive to PEG-induced water stress (*OsMYB48–1* ↑↓), H_2_O_2_ (*OsMYB48–1* ↑), drought (*OsMYB48–1* ↑, *OsMYB2* ↑; *OsWRKY90* ↑), dehydration (*OsMYB48–1* ↑), desiccation (*OsMYB2* ↑), salinity (*OsMYB48–1* ↑, *OsMYB2* ↑) and cold (*OsMYB48–1* ↑, *OsMYB2* ↑). Other stress responses include pathogen attack (*OsWRKY90* ↑), wounding (*OsWRKY90* ↑), iron toxicity (*OsWRKY90* ↑) and senescence *(OsWRKY90* ↑) (Ricachenevsky, Sperotto, Menguer, and Fett, 2010) (Katiyar et al., 2012; Xiong et al., 2014). *OsMYB48–1* also plays a positive role in drought and salinity tolerance by regulating ABA synthesis (Xiong et al., 2014).

The other important set of genes is GA-responsive, including *OsSUB1C* (↑) and the Zn finger *OsBBX30* (LOC_Os12g10660) mostly expressed in vegetative tissues and mature panicles (↑), the B-Box factors *OsBBX1 and OsBBX7* (↑), the WRKY gene *OsWRKY62* (LOC_Os09g25070), highly expressed in mature leaves and young roots (↑), *OsWRKY71/OsEXB1* (LOC_Os02g08440) (↓), *OsANAC34* (↓) and two MYB TF genes (*OsMYB2* and LOC_Os03g04900) (↓). Expression of these TFs was also responsive to light (*OsBBX30* ↑), auxin (*OsBBX30* ↑, *OsBBX1* ↑), cytokinin (*OsBBX30* ↑, *OsBBX1* ↑), IAA (*OsWRKY62* ↑), SA (*OsWRKY62* ↑, *OsWRKY71*↑), JA (*OsWRKY71*↑), ABA (*OsWRKY71*↑↑), desiccation (*OsWRKY62* ↑, MYB ↑), cold (*OsWRKY62* ↓), osmotic stress (*OsWRKY62* ↑), salinity (MYB ↑), and pathogen attack (*OsWRKY62* ↑) (Ramamoorthy et al., 2008; Shankar et al., 2016; Xiang et al., 2017). *OsBBX7* (LOC_Os02g49230) is highly expressed in leaves, hull and endosperm and is light-responsive. It displays a diurnal expression pattern, i.e. upregulated at night under short-day conditions and high expression during light compared to dark under long-day conditions (Huang et al., 2012). *OsWRKY62* was also observed to be non-responsive to ABA treatment and therefore likely acts in an ABA-independent signalling pathway. *OsWRKY71* is a general stress-responsive gene and was reported to be upregulated by submergence in M202 and dehydration in IR64. However, this response was not observed in M202-Sub1 and IR64-Sub1 (Fig. 2) (Fukao et al., 2011). This gene is also involved in desiccation and salt stress responses in the rice variety N22 (Shankar et al., 2016) and overexpression of *OsWRKY71* in rice enhances tolerance to cold (Kim et al., 2016) and resistance to Xanthomonas (Liu, Bai, Wang, and Chu, 2007; Berri et al., 2009; Chujo et al., 2013). The MYB TF gene shares high sequence similarity with MULTIPASS (*OsMPS*), which regulates plant growth via expansins and was shown to be downregulated by GA and auxin but upregulated by ABA and cytokinin (Schmidt et al., 2013).

The other TF genes have not been reported to be ABA- or GA-responsive and/or responsive to submergence. *OsBBI1* (LOC_Os06g03580) and *OsONAC7* (LOC_Os11g05614) have been associated with biotic stress response. *OsBBI1* encodes a RING finger protein with E3 ligase activity, which modifies cell wall defence and confers broad-spectrum disease resistance to blast fungus (*Magnaporthe oryzae)*. Expression of *OsBBI1* is also induced by the rice blast fungus, benzothiadiazole and SA (Li et al., 2011). *OsONAC7* is expressed and highly upregulated in rice stripe virus (RSV) and tungro virus (RTSV) infected plants (Nuruzzaman et al., 2010). The NAC TF gene *OsSNAC* (LOC_Os07g37920) was shown to be downregulated under drought stress in the intolerant variety Nipponbare (Nuruzzaman et al., 2010) but upregulated under desiccation stress in the rainfed varieties N22 and Pokkali (Shankar et al., 2016).

Limited information is available for the remaining genes downregulated in IR64. An *in-silico* study proposed B3 domain factor LOC_Os08g06120 to colocalize with a QTL for seed width (Peng and Weselake, 2013). The PHD Zn finger gene *OsMYB7* (LOC_Os08g43550) and the CCT motif orphan gene (LOC_Os02g05470) have no defined function but were both downregulated under desiccation stress in IR64 (Shankar et al., 2016). *OsWRKY19* (LOC_Os05g49620) was shown to be constitutively low expressed across different tissues and was upregulated by IAA and SA with no clear involvement in any stress response (Ramamoorthy et al., 2008).

### *SUB1A* promoter analysis

**Fig. 5:**
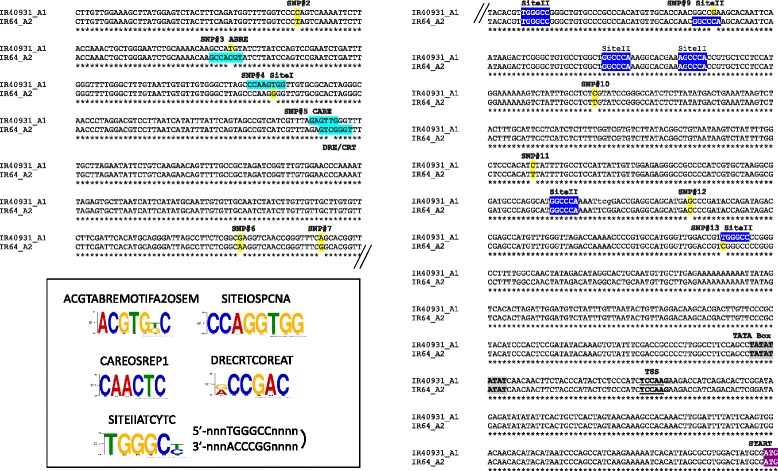
Comparison between the *SUB1A-1 and SUB1A-2* upstream region. The *SUB1A-1* and *SUB1A-2* upstream regions were aligned and analysed to identify the promoter region and allele-specific putative *cis*-elements constituted by the thirteen single nucleotide polymorphisms (SNPs). Annotation of the putative *cis*-elements is indicated in the figure and explained in detail in the text. Other relevant promoter elements are additionally indicated. TSS, transcription start site. The complete alignment is provided in Additional file 1: Figure S1

A comparative sequence analysis of the promoter region of the *SUB1A-1* and *SUB1A-2* alleles (Additional file 2: Table S5) revealed the presence of 13 SNPs. Out of these, 5 SNPs gave rise to allele-specific putative *cis*-regulatory elements that could be targets of upstream TFs. (Fig. 5; Additional file 3: Figure S2).

In the *SUB1A-1* allele, SNP4, SNP5, and SNP13 create putative motifs (Fig. 5). SNP4 constitutes a Site I (SITEIOSPCNA) motif that resembles the G-box and was discovered in the rice *PROLIFERATING CELL NUCLEAR ANTIGEN* (*PCNA*) promoter. Promoter deletion studies had shown that the Site I element is a transcriptional activation site of PCNA and that it is essential for meristematic tissue-specific expression (Kosugi, Suzuka, and Ohashi, 1995). SNP5 resulted in two distinct *cis*-elements in the two *SUB1A* alleles (see below) creating a CAREOSREP1 motif in *SUB1A-1*. This motif was shown to confer GA-mediated increased expression of a proteinase gene in rice seeds (Sutoh and Yamauchi, 2003). SNP13 creates the other constituent of the Site I bipartite motif, i.e., the Site II element (SITEIIATCYTC). This motif was also formed by SNP9 in the *SUB1A-2* allele (see below). In addition to *PCNA*, Site II elements are present in the promoters of many Arabidopsis cell cycle, mitochondrial, ribosomal and cytochrome genes (Tremousaygue et al., 2003; Welchen and Gonzalez, 2005). They are also overrepresented in the promoters of genes encoding nuclear components of the oxidative phosphorylation complex in Arabidopsis and rice (Welchen and Gonzalez, 2005; Welchen and Gonzalez, 2006). Promoter::GUS studies showed that the Site II motif is sufficient to mediate gene expression in shoot and root meristems, as well as in anthers (Kosugi and Ohashi, 1997). It is recognised by TCP and bHLH TFs, which play crucial roles in hormone response pathways, light and abiotic stress responses (Danisman, 2016). Importantly, the position of the Site II element specific to the *SUB1A-1* allele is closer to the transcription start site (TSS), which was shown to be the more active position, thus makes this element more relevant in *SUB1A-1* allele compared to *SUB1A-2*, where it is located further upstream (Tremousaygue et al., 2003; Welchen and Gonzalez, 2006).

The *Sub1A-2* allele had three SNPs (SNP3, SNP5, SNP9) creating allele-specific putative *cis*-regulatory elements. SNP3 generates an ABRE motif (ACGTABREMOTIFA2OSEM) in the negative strand, a motif present in many ABA-responsive genes (Hattori, Totsuka, Hobo, Kagaya, and Yamamoto-Toyoda, 2002; Narusaka et al., 2003). SNP5 constitutes the core of the DRE/CRT (dehydration-responsive element/C-repeat; DRECRTCOREAT) motif present in many drought-responsive Arabidopsis and rice genes (Dubouzet et al., 2003; Qin et al., 2004; Diaz-Martin, Almoguera, Prieto-Dapena, Espinosa, and Jordano, 2005; Skinner et al., 2005; Suzuki, Ketterling, and McCarty, 2005). As is the case for SNP13 in the *SUB1A-1* allele, SNP9 gave rise to a Site II element (SITEIIATCYTC) but was located further upstream of the TSS as mentioned above.

A third Site II element is present in both alleles (Fig. 5). In addition to Site I and Site II elements, a telo-box (5’-AAACCCTAA-3′) was identified in the *PCNA* promoters and shown to act as a transcriptional activator (Tremousaygue et al., 2003). In the *SUB1A* alleles, no perfect match for the telo-box could be found, however, two truncated telo-box motifs (5’-ACCCTA-3′ and 5’-AAACCCT-3′) are present near the *SUB1A-2* and *SUB1A-1* specific Site II elements, respectively (Fig. 5).

### Promoter analysis of the DEGs

The analysis of the 2Kb upstream regions of the DEGs identified 32 distinct motifs present in at least one of the genes (Table 3). These motifs can be categorized into four groups based on their relatedness to hypoxia & light, energy & sugar metabolism, transcription & cell division, and hormones & stress.

**Table 3:** Putative *cis*-elements identified in differentially expressed TF genes

CATERGORY	MOTIF	IR64 down (#27 genes)	IR64-Sub1 down (#7 genes)	IR64-Sub1 up (#13 genes)
Hypoxia & light	GT1CONC4:C36SENSUS	26	7	13
	ANAERO2CONSENSUS	18	6	8
	ANAERO1CONSENSUS	16	5	8
	ANAERO3CONSENSUS	9	2	4
	ANAERO4CONSENSUS	2	0	1
Energy	PYRIMIDINEBOXOSRAMY1A	24	7	10
	TATCCAOSAMY	17	4	12
	CGACGOSAMY3	16	7	8
	TATCCAYMOTIFOSRAMY3D	9	1	8
	CAREOSREP1	23	5	7
	SITEIIATCYTC	21	5	10
	SITEIIBOSPCNA	1	0	0
	SITEIIAOSPCNA	0	0	1
	SITEIOSPCNA	2	0	2
	AACACOREOSGLUB1	13	3	3
	PROLAMINBOXOSGLUB1	5	1	5
	GCN4OSGLUB1	8	1	0
	ACGTOSGLUB1	4	2	3
	GARE1OSREP1	4	4	1
	GARE2OSREP1	0	3	2
	BP5OSWX	3	2	2
Transcription & cell devision	BIHD1OS	25	7	12
	TATABOXOSPAL	16	4	7
	TATABOX1	2	1	0
	E2FCONSENSUS	13	1	10
	E2F1OSPCNA	2	0	0
	LEAFYATAG	3	0	4
	WUSATAg	6	0	1
Hormones and stress	DRECRTCOREAT	17	6	7
	ACGTABREMOTIFA2OSEM	9	4	5
	ABREOSRAB21	6	4	2
	IRO2OS	3	1	0

Under submergence, plants are exposed to hypoxia and low light conditions and in agreement with this, four anaerobic consensus motifs (ANAERO1–4) and the light response motif GT1CONC4:C36SENSUS were found in most DEGs. The anaerobic consensus motifs were previously identified in genes involved in the anaerobic fermentative pathway (Mohanty, Krishnan, Swarup, and Bajic, 2005). The GT1 motif has been found in light-regulated genes and is known to stabilize the TATA box complex (Terzaghi and Cashmore, 1995; Villain, Mache, and Zhou, 1996; Buchel, Brederode, Bol, and Linthorst, 1999; Le Gourrierec, Li, and Zhou, 1999; Zhou, 1999).

Sixteen of the identified motifs were related to energy and sugar metabolism, and studies associated with some of these motifs are detailed below. RAMY motifs, identified in alpha-amylase genes, were highly represented in all four groups of DEGs (up and downregulated DEG in IR64 and IR64-Sub1). The RAMY3D motif (TATCCAY) was found in the rice *RAmy3D* alpha-amylase gene promoter and is responsible for sugar repression (Toyofuku, T-a, and Yamaguchi, 1998). The AMY3 motif (CGACGO) is present in the GC-rich regions of amylase genes and functions as a coupling element for the G-box element (Hwang, Karrer, Thomas, Chen, and Rodriguez, 1998). The other AMY motif (TATCCA) is a binding site for *OsMYBS1*, *OsMYBS2* and *OsMYBS3* TFs, which mediate sugar and hormone regulation of alpha-amylase gene expression (Lu, Lim, and Yu, 1998; Lu, Ho, Ho, and Yu, 2002; Chen, Chiang, Tseng, and Yu, 2006). GARE and CARE motifs are necessary for GA regulation of alpha-amylases genes (Sutoh and Yamauchi, 2003) and the BP5OSWX motif acts as a transcriptional activator in the waxy gene (Zhu, Cai, Wang, and Hong, 2003). The GARE motif was present in about 50% of the IR64-Sub1 upregulated genes and less frequent (15%) in the downregulated genes in both genotypes (Table 3). The GARE and RAMY1A (PYRIMIDINEBOX) motifs participate in sugar repression and are present in the promoter of the rice alpha-amylase gene *RAmy1A* and the barley alpha-amylase gene *Amy2/32b,* both of which are GA inducible (Morita et al., 1998; Mena, Cejudo, Isabel-Lamoneda, and Carbonero, 2002). The four identified GLUB1 motifs have been associated with the rice glutelin gene *OsGluB-1*. They are related to energy metabolism and are proposed to control endosperm-specific expression (Washida et al., 1999; Wu, Washida, Onodera, Harada, and Takaiwa, 2000; Qu, Xing, Liu, Xu, and Song, 2008). Glutelins are primary energy storage proteins and the relevance of these motifs remains to be assessed with respect to submergence response. Site II motifs as detailed above, were also found in most DEGs.

Seven of the identified motifs have been related to transcription (E2F, EF1, and TATA Box), meristems (LEAFYATAG and WUSATAg) and homeotic development (BIHD1OS). The E2F binding site was present in 80% of the IR64-Sub1 upregulated genes and less frequent in the downregulated genes (IR64–50% and IR64-Sub1–10%). E2F TFs are regulators of the cell cycle and can act as transcriptional activators or repressors (Vandepoele et al., 2005). The meristem motifs were rare compared to BIHD1OS, which was present in most DEGs. The BIHD1OS motif has been reported to be the binding site for *OsBIHD1*, a rice BELL homeodomain TF that integrates ethylene and BR hormonal responses (Liu et al., 2017).

Four of the DEG motifs were associated with hormone and stress responses. The drought-related DRE/CRT and ABA-responsive ABRE motifs were most frequent (Dubouzet et al., 2003; Qin et al., 2004; Diaz-Martin et al., 2005; Skinner et al., 2005; Suzuki et al., 2005), whereas the Fe starvation related motif IRO2 (Ogo et al., 2006) was present in only about 10% of the genes. Amongst the groups of DEGs, DRE/CRT and ABRE motifs were most common in the IR64-Sub1 downregulated genes (60–80%) compared to the other two groups (Table 3).

## Discussion

All *SUB1A*-related studies so far have been conducted using contrasting genotypes with and without the *SUB1A* gene and, therefore, limited information is available on the differential effect of the two alleles. As a first step, the objective of this study was therefore to assess if different sets of TF genes are regulated by the strong *SUB1A-1* allele, as present in IR64-Sub1, and the weaker *SUB1A-2* allele, naturally present in the wild-type IR64. An earlier study on a range of rice genotypes had shown that both alleles are highly expressed in nodes of submerged plants, however, differences in the expression level in internodes were most pronounced and correlated best with submergence tolerance (Singh et al., 2010). Therefore, internode tissue was used for this comparative study.

The qPCR analysis of the 2487 TF genes revealed a relatively small number (46) of TF genes that were significantly differentially expressed under submergence. Interestingly, the predominant transcriptional response was reduced expression and only thirteen genes, all in IR64-Sub1, were upregulated under submergence. This, per se, might at least partly explain the higher tolerance level of IR64-Sub1. Further to this, with the exception of an uncharacterized MYB factor, distinct sets of TFs were identified in IR64 and IR64-Sub1, suggesting that the two genotypes employ different regulatory pathways leading to different levels of tolerance.

### Association between submergence responsive DEGs and plant hormones

Nineteen TF genes were specifically submergence-responsive in IR64-Sub1, making them candidates to explain the higher tolerance of IR64-Sub1 compared to IR64. The upregulated genes were previously reported in studies on drought, salt and other abiotic stresses, but not with respect to submergence stress (Additional file 2: Table S7). In agreement with our data, the *OsbZIP4* and the Zn-finger gene LOC_Os07g43740 were found to be upregulated under anoxia (Ham et al., 2013; Shankar et al., 2016).

Available data on the hormonal response of some of these genes (*OsHsfC2a, OsBBX11, OsBBX24, OsWRKY76 and OsTIFY11a*) suggest that they are part of the ABA/GA regulatory pathway (Fig. 4). For instance, one of the six upregulated MYB factors (LOC_Os08g06110) was reported to reduce seed dormancy in a rice mutant through ABA-GA antagonism (Wu et al., 2016). *OsHsfC2a* is downregulated by ABA, while, GA causes downregulation of *OsBBX11* and *OsBBX24* (Huang et al., 2012). Additionally, *OsBBX11* is regulated by light, which plays a critical role in the submergence response (Das et al., 2009).

Among the genes downregulated in IR64-Sub1 were two AP2-type TFs (*OsERF136, OsDREB2b*), both of which are submergence-responsive in M202 and M202-Sub1 (Jung et al., 2010). Since the wild-type genotype M202 naturally lacks *SUB1A,* the two AP2 genes might act in a different pathway, independent of *SUB1A*. *DREB2b* has also been reported to function as a transcriptional activator under low- and high-temperature stress and it enhances drought tolerance when overexpressed (Matsukura et al., 2010; Todaka et al., 2012). Similarly, *SUB1A* has been implicated with enhanced recovery growth after severe drought (Fukao et al., 2011) and submerged plants generally survive longer in cooler water (Das et al., 2009). These similarities between *SUB1A* and *DREB2b* make the latter an interesting candidate gene for integrating these signal responses.

In contrast to IR64-Sub1, the DEGs in IR64 were all downregulated (Fig. 5). The most highly downregulated gene encodes an uncharacterized protein, with putative Jas- and PHD Zn-finger domains, suggesting that it may be a part of the JA signalling pathway and/or a regulator of development. The second most highly responsive gene was *OsSUB1B*, followed by WRKY and additional ERF/AP2 factors, including *OsSUB1C*. Interestingly, both *OsSUB1B* and *OsSUB1C* are, despite also in IR64-Sub1, only differentially expressed (downregulated) in IR64. *OsSUB1B* is non-responsive to GA and ethylene and no tolerant-specific allele has been identified for this gene (Xu et al., 2006). A clear downregulation of *OsSUB1B* under submergence in IR64 justifies more in-depth analysis of its function and regulation of expression. In contrast, tolerance-specific alleles for *OsSUB1C* are known and its induction by GA and ethylene specifically in M202-Sub1 has been documented (Fukao et al., 2006; Xu et al., 2006). These studies also indicated that *SUB1A-1* might be a negative regulator of *OsSUB1C*. Downregulation of *OsSUB1C* in IR64 suggests that the *OsSUB1A-2* allele can likewise act as a negative regulator of *OsSUB1C*, directly or indirectly.

The three AP2/ERF genes downregulated in IR64 (*OsERF067, OsERF066, OsERF922*) had previously been shown to be upregulated in M202-Sub1 under submergence (Jung et al., 2010). This was not observed in IR64-Sub1 in this study, however, their downregulation in IR64 might at least partly explain a lower level of submergence tolerance. *OsERF922* may be of particular interest here since this gene acts as a transcriptional activator and modulator of ABA levels (Liu et al., 2012).

The *BBX1* gene had previously been shown to be submergence induced in the variety M202, which lacks the *SUB1A* gene (Fukao et al., 2011). This is in contrast to the observed downregulation of *BBX1* in IR64 in our study, suggesting that *BBX1* might be under the control of the *SUB1A-2* allele contributing to the higher tolerance level of IR64 compared with M202.

As was the case for IR64-Sub1, a number of the IR64 downregulated DEGs have been associated with GA and ABA regulation. Among the GA-responsive genes, the MYB TF (LOC_Os03g04900) might be of particular interest as it is similar to *OsMPS*, which acts as a regulator of plant growth by suppressing expansin genes and is downregulated by GA and upregulated by ABA treatment (Schmidt et al., 2013). Suppression of expansins has previously been shown to be a critical component of the *SUB1A*-mediated quiescence response and it will be interesting to assess if this MYB factor functions in a way similar to *OsMPS*.

Genes upregulated by ABA (*OsMYB2*, *OsWRKY90*, *OsERF922 and OsMYB48–1*) activate the ABA synthesis pathway and *OsMYB48–1*, specifically, has been reported to induce other ABA-responsive genes under drought (Xiong et al., 2014). On the other hand, four genes (*OsSUB1C*, *OsBBX1, OsBBX30, OsWRKY62*) have antagonistic effects to ABA and respond positively to GA (Ramamoorthy et al., 2008; Huang et al., 2012; Shankar et al., 2016). The fact that all of these genes are downregulated in IR64 under submergence suggests that at least part of the GA signalling pathway is inactivated, as required for submergence tolerance. That these genes have not identified as differentially expressed in IR64-Sub1 is not necessarily a contradiction but instead suggests that submergence tolerance might be related to low or neutral expression of these genes.

### Role of iron uptake and JA in submergence tolerance

Iron solubility and availability in plants is altered based on soil pH and submergence conditions and can result in excess iron availability. Difficulties in iron uptake cause chlorosis whereas iron overload or increased uptake leads to oxidative stress and permanent cell and tissue damage (Zheng 2010). One of the four TFs that showed a constitutive difference between the two genotypes, *OsIRO2,* was constitutively lower expressed in IR64-Sub1 (Fig. 2a). *IRO2* has so far only been described in relation to Fe and it was shown to be specifically induced by Fe deficiency (Ogo et al., 2006; Kobayashi et al., 2007). Although the specific role during submergence remains to be determined, the higher expression of the *IRO2* in IR64 might suggest a higher Fe uptake in this cultivar resulting in oxidative stress and cell damage.

As expression of *IRO2* is not induced by submergence, a specific role for *IRO2* is perhaps unlikely, however, it might be possible that the *SUB1A-2* allele specifically acts as an enhancer of *OsIRO2* thereby causing a secondary, negative effect under submergence. It will, therefore, be interesting to compare the performance of IR64 and IR64-Sub1 under Fe deficiency conditions and quantify plant iron content.

Five of the other TF genes identified in this study (*OsTIFY11e, OsTIFY11d, OsWRKY76, OsTIFY11a, OsWRKY71*) were reported to be upregulated by JA and downregulated by ABA (Ye et al., 2009; Ranjan et al., 2015; Yang et al., 2015; Shankar et al., 2016). Additionally, *WRKY76* and *WRKY71* are also upregulated by GA (Ramamoorthy et al., 2008; Shankar et al., 2016). This is well in support of the finding that fine-tuning of GA and ABA levels under submergence is a major component of regulating elongation growth in rice (Fukao et al., 2006; Fukao and Bailey-Serres, 2008) and studies on Arabidopsis have further shown that JA plays a crucial role in protecting plants during post-submergence re-oxygenation (Yuan et al., 2017). The genes identified in this study might therefore play a role in integrating various hormonal signals and analysing their expression in IR64 and IR64-Sub1 plants upon de-submergence will provide more insights into the role of *SUB1A-2* during recovery growth.

A significant number of DEGs controlled by submergence have also been described as cold stress responsive. In addition, it has been shown that plant survival in cooler submergence water is higher (Das et al., 2009). Therefore, higher expression of these cold-induced genes in IR64-Sub1 may contribute to its higher tolerance level. For the genes listed in Additional file 2: Table S7, cold responsiveness was also observed in M202 and M202-Sub1 (Jung et al., 2010).

### Putative *cis*-regulatory elements

The diversity of the putative regulatory *cis-*elements identified in the promoters of the DEGs (Table 3) is well in agreement with the diverse environmental stimuli associated with submergence requiring plants to adjust to low oxygen, light and temperature, alter their energy and sugar metabolism, cell divisions, as well as hormone-regulated growth. For instance, the light-responsive motif (GT1CONC4:C36SENSUS) occurred in all genes except one, suggesting that low-light is an integral signal that can trigger both, up- and downregulation of genes. Likewise, the majority of genes appears to be responsive to hypoxia, as indicated by the presence of the anaerobic consensus motifs, ANAERO1CONSENSUS and ANAERO2CONSENSUS, as well as the PYRIMIDINEBOXOSRAMY1A, found in alpha-amylase genes. The AMY motif (TATCCA) appears to be more frequent in IR64-Sub1 downregulated genes, whereas the AMY3 motifs (CGACG), which functions as a G-box coupling element, is more prevalent in upregulated genes. The former is the binding site of *OsMYBs,* which mediate sugar and hormone regulation of alpha-amylase genes (Lu et al., 1998; Lu et al., 2002; Chen et al., 2006). Since maintenance of a higher level of soluble sugars has been implicated with tolerance and better recovery growth (Singh et al., 2001; Das et al., 2005; Fukao et al., 2006) these TF genes might be part of the regulatory pathway controlling sugar consumption under submergence. Despite the importance of ABA in submergence tolerance, surprisingly few genes had ABRE elements. In contrast, the DRE/CRT element was more frequent, which, in conjunction with the identified differential expression of *DREB2b* (Matsukura et al., 2010; Todaka et al., 2012), reinforces a possible relationship between submergence and drought tolerance (Fukao et al., 2011).

Among the most interesting elements identified in the promoter analysis is the bi-partite Site II element. This motif was frequently present in the DEGs and also distinguished the *SUB1A-1* and *SUB1A-2* alleles at the SNPs (Fig. 5). Since this motif has been implicated with meristem-specific expression (Tremousaygue et al., 2003; Welchen and Gonzalez, 2006) its presence might indicate that control of cell division, in addition to elongation growth, is important for tolerance. This is supported by promoter::GUS reporter studies that showed specific *SUB1A-1* expression in the base of growing leaves (Singh et al., 2010). It will be interesting to study this motif in more detail and to determine if the position closer to the TTS of the *SUB1A-1-*specific Site II element enhances the capacity of tolerant plants to suppress cell division under submergence. In relation to this, it is relevant that the two *SUB1A* alleles are also distinct in their coding region for a MAPK3 phosphorylation target site, which is specifically present in SUB1A-1 (Singh and Sinha, 2016). The same study also showed that both, SUB1A-1 and SUB1A-2, physically interact with MAPK3 but that the interaction with SUB1A-2 was weaker. Thus, SUB1A-2 would still be a component of this regulatory pathway but might be less efficient. It will indeed be interesting to investigate the role of SUB1A in regulating cell division in more detail and to establish whether SUB1A-2 binds to the *MAPK3* promoter, as was shown for SUB1A-1 (Singh and Sinha, 2016).

## Conclusion

This study identified distinct sets of submergence-responsive transcription factor genes in IR64 and IR64-Sub1 which might at least partly explain differences in the level of tolerance between the two genotypes. These genes should now be assessed for putative additive effects with *SUB1A* and their potential to enhance tolerance. The promoter analysis identified allele-specific site II elements, which should be studied in more detail to establish if *SUB1A* directly or indirectly suppresses cell division, in addition to its documented role in suppressing cell elongation.

## Additional files

Additional file 1: Figure S1. The submergence tolerance locus Sub1 in rice. The Sub1 locus is located on rice chromosome 9 with a variable number of *SUB1* genes. In different rice genotypes, *SUB1A* is either absent or present as different allele. (**a**). Introgression of the Sub1 locus into the rice variety IR64 (IR64-Sub1) enhanced survival after complete submergence. (**b**). The photo shows the IRRI demonstration field plot in the Philippines. Phenotyping for submergence tolerance can also be conducted by submerging plants grown in trays (inlay). The *SUB1A-1* and *SUB1A-2* alleles are both highly expressed in nodes of submerged plants but *SUB1A-1* expression is higher in internodes. (**c**). Schematic illustration based on (Singh et al., 2010). (PPTX 3066 kb)

Additional file 2: **Table S1.** List of genes represented in Rice TF Primer Platform (Caldana et al., 2007). Complete list of 2508 TF genes, their primer sequences and their corresponding PCR efficiencies. Genes were presented with version 2.0 and version 5.0 RGAP genome annotations. **Table S2.** Transcription factor genes with and without annotations in version 5.0 and 7.0 of RGAP Pseudomolecule. **Table S3.** Expression profile of transcription factor genes in IR64 control and stress-treated plants upon submergence. Recorded Ct, R^2^, Efficiency and ΔCt values for all reactions. The list of used reference genes and their distribution on the plates is also given. **Table S4.** Expression profile of transcription factor genes in IR64-Sub1 control and stress-treated plants upon submergence. Worksheets record Ct, R^2^, Efficiency and ΔCt values for all reactions. The list of used reference genes and their distribution on the plates is also given. **Table S5.** Promoter sequences of *SUB1A-1* and *SUB1A-2*. **Table S6.** Moderated t-tests results from LIMMA package for identifying differentially expressed genes. *P*-values were recorded and FDR corrections were also performed. **Table S7.** Details about the list of differentially expressed genes and their putative functions. (DOCX 29 kb)

Additional file 3; Figure S2. Sequence alignment of the *SUB1A-1* and *SUB1A-2* upstream promoter regions. 2 kb upstream of the start site (promoter regions) were analysed for *SUB1A-1* and *SUB1A-2* alleles and putative *cis*-regulatory elements were identified. (XLSX 3.76 kb)
