# Supplementary material for: Allelic variants of OsSUB1A cause differential expression of transcription factor genes in response to submergence in rice
Source: Rice (N Y). 2018 Jan 8;11:2. doi: 10.1186/s12284-017-0192-z (PMC5758481; doi:10.1186/s12284-017-0192-z)
Supplement: Supplementary file 1 — The submergence tolerance locus Sub1 in rice. The Sub1 locus is located on rice chromosome 9 with a variable number of SUB1 genes. In different rice genotypes, SUB1A is either absent or present as different allele. (a). Introgression of the Sub1 locus into the rice variety IR64 (IR64-Sub1) enhanced survival after complete submergence. (b). The photo shows the IRRI demonstration field plot in the Philippines. Phenotyping for submergence tolerance can also be conducted by submerging plants grown in trays (inlay). The SUB1A-1 and SUB1A-2 alleles are both highly expressed in nodes of submerged plants but SUB1A-1 expression is higher in internodes. (c). Schematic illustration based on (Singh et al., 2010). (PPTX 3066 kb) [file 12284_2017_192_MOESM1_ESM.pptx]

## Slide 1
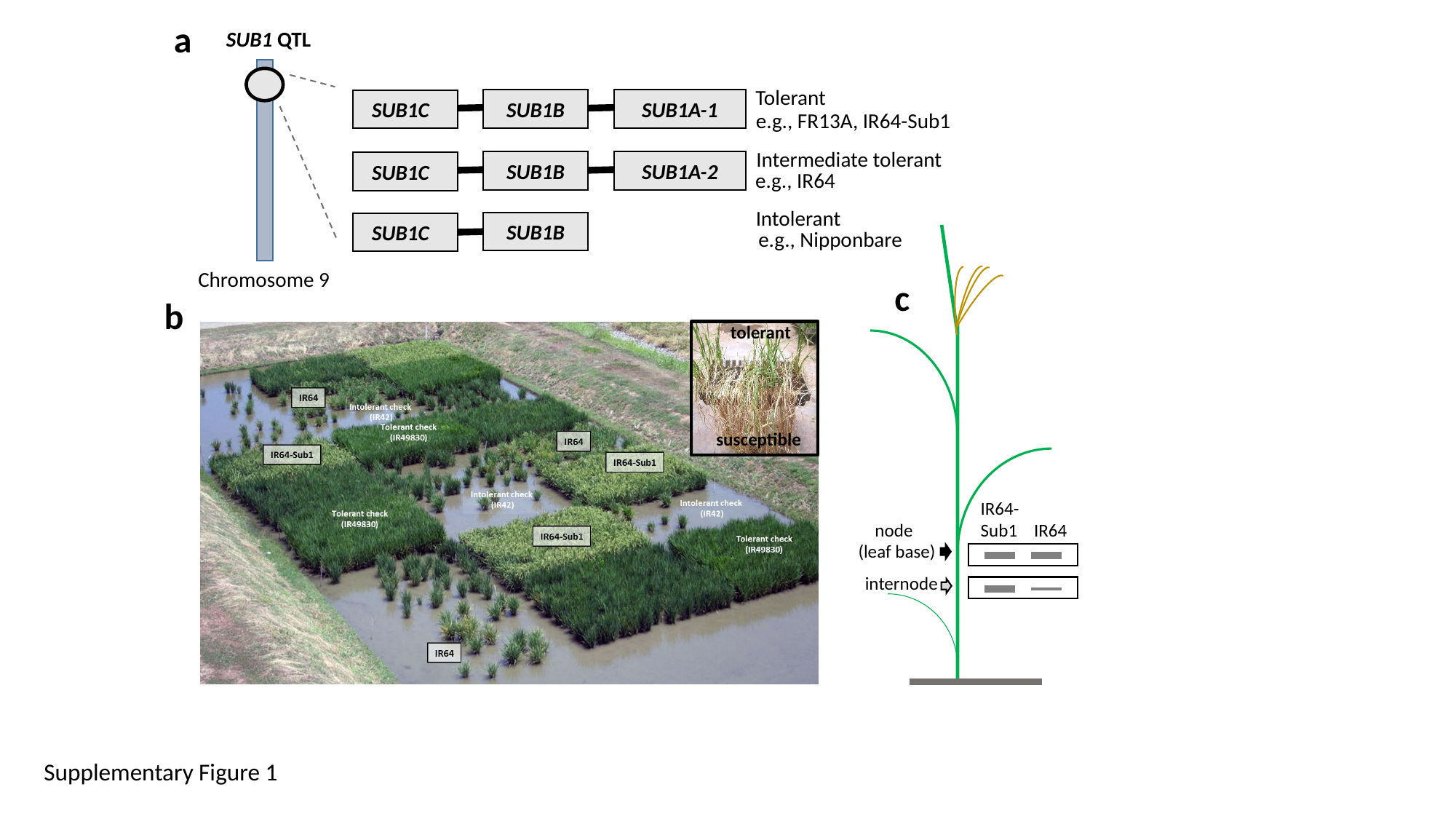

a
SUB1 QTL
Chromosome 9
Tolerant
e.g., FR13A, IR64-Sub1
SUB1B
SUB1A-1
SUB1C
Intermediate tolerant
e.g., IR64
SUB1B
SUB1A-2
SUB1C
Intolerant
e.g., Nipponbare
SUB1B
SUB1C
 node
(leaf base)
internode
IR64-
Sub1 IR64
c
b
tolerant
susceptible
Supplementary Figure 1
