# Supplementary material for: Allelic variants of OsSUB1A cause differential expression of transcription factor genes in response to submergence in rice
Source: Rice (N Y). 2018 Jan 8;11:2. doi: 10.1186/s12284-017-0192-z (PMC5758481; doi:10.1186/s12284-017-0192-z)
Supplement: Supplementary file 2 — Table S1. List of genes represented in Rice TF Primer Platform (Caldana et al., 2007). Complete list of 2508 TF genes, their primer sequences and their corresponding PCR efficiencies. Genes were presented with version 2.0 and version 5.0 RGAP genome annotations. Table S2. Transcription factor genes with and without annotations in version 5.0 and 7.0 of RGAP Pseudomolecule. Table S3. Expression profile of transcription factor genes in IR64 control and stress-treated plants upon submergence. Recorded Ct, R2, Efficiency and ΔCt values for all reactions. The list of used reference genes and their distribution on the plates is also given. Table S4. Expression profile of transcription factor genes in IR64-Sub1 control and stress-treated plants upon submergence. Worksheets record Ct, R2, Efficiency and ΔCt values for all reactions. The list of used reference genes and their distribution on the plates is also given. Table S5. Promoter sequences of SUB1A-1 and SUB1A-2. Table S6. Moderated t-tests results from LIMMA package for identifying differentially expressed genes. P-values were recorded and FDR corrections were also performed. Table S7. Details about the list of differentially expressed genes and their putative functions. (DOCX 29 kb) [file 12284_2017_192_MOESM2_ESM.docx]

IR40931_A1 GGTAATGAGGAAAGAGAAGAAAGACAAAGAGGAAAAATACCTATCTATAGCTCCATGCAT

IR64_A2 GGTAATGAGGAAAGAGAAGAAAGACAAAGAGGAAAAATACCTATCTATAGCTCCATGCAT

************************************************************

IR40931_A1 GCTTGAGGAATGTTTAATTGACCAAGCGCCTACAATTTCTGAAGATGAGCAAAAAAAAAG

IR64_A2 GCTTGAGGAATGTTTAATTGACCAAGCGCCTACAATTTCTGAAGATGAGCAAAAAAAAAG

************************************************************

IR40931_A1 AAATGATAATGGTGCTACAACTACTCAAGGTATGCATAGTTATGATGTGCCAACTATGAC

IR64_A2 AAATGATAATGGTGCTACAACTACTCAAGGTATGCATAGTTATGATGTGCCAACTATGAC

************************************************************

IR40931_A1 CACTACTTATGCTACATTAGAGAAACCCATAGTGGAAACAATTGTTGAAATACCTTTGTC

IR64_A2 CACTACTTATGCTACATTAGAGAAACCCATAGTGGAAACAATTGTTGAAATACCTTTGTC

************************************************************

**SNP#1**

IR40931_A1 ACAAAACAATTTGTTTGATGTTTCTTGTGATAAAGAAGAGTTGTGTGATGCTTCATTTAT

IR64_A2 ACAAAACAATTTGTTTGATGTTTCTTGTGATAAAGAAAAGTTGTGTGATGCTTCATTTAT

************************************* **********************

IR40931_A1 ATCCTTGCCACAACTAGTGAATGAACATGTTAGTTCTATAGTAGAGCCACCATGTGTTCA

IR64_A2 ATCCTTGCCACAACTAGTGAATGAACATGTTAGTTCTATAGTAGAGCCACCATGTGTTCA

************************************************************

IR40931_A1 GTTTAAACATGTTATTCACATTGCTAGCGAGAATGAAGAGCTTAAATTACTATCTTTTTT

IR64_A2 GTTTAAACATGTTATTCACATTGCTAGCGAGAATGAAGAGCTTAAATTACTATCTTTTTT

************************************************************

IR40931_A1 AAATACTTGGGGTTATATTCAATTTGATGATCTTTGTCCACTCAACTGTTTAGAGGAGAA

IR64_A2 AAATACTTGGGGTTATATTCAATTTGATGATCTTTGTCCACTCAACTGTTTAGAGGAGAA

************************************************************

IR40931_A1 ACTACTTGCTAGATTTGAATTGCCATGCCCTTCCGATGTGATTTTTCATGTTATTGGCAA

IR64_A2 ACTACTTGCTAGATTTGAATTGCCATGCCCTTCCGATGTGATTTTTCATGTTATTGGCAA

************************************************************

IR40931_A1 CTATGATAGTAAAGGAGAATATAATATGCATCGAGTATATATTTGTTCGAATTTGACTAT

IR64_A2 CTATGATAGTAAAGGAGAATATAATATGCATCGAGTATATATTTGTTCGAATTTGACTAT

************************************************************

IR40931_A1 ACCTCCTTTTCATGATGAAATGTATTGTCTAGAGGACCGCATGCACATAAGTAATTCTCT

IR64_A2 ACCTCCTTTTCATGATGAAATGTATTGTCTAGAGGACCGCATGCACATAAGTAATTCTCT

************************************************************

IR40931_A1 ATATAGTTCCTCTAGTTGCTTAAATGAGTTACATGTTGGTTTGTAAGAAAGGGAGTGTTG

IR64_A2 ATATAGTTCCTCTAGTTGCTTAAATGAGTTACATGTTGGTTTGTAAGAAAGGGAGTGTTG

************************************************************

IR40931_A1 TGGTTTTTCGACTACAAAGATTTCAGGATCCAACCATATGGCCAGCAAAAAATGTTCCTC

IR64_A2 TGGTTTTTCGACTACAAAGATTTCAGGATCCAACCATATGGCCAGCAAAAAATGTTCCTC

************************************************************

IR40931_A1 ACCGGATGTTGTGATAGATAAAGATAAGTTCAATACGTTTCATGACAAGGTGAAATCGAG

IR64_A2 ACCGGATGTTGTGATAGATAAAGATAAGTTCAATACGTTTCATGACAAGGTGAAATCGAG

************************************************************

IR40931_A1 GGCGGTTTCCAATCAAGAAAGGGAGGATGATGAGGACACGACTAGCTCGGATATAACCAT

IR64_A2 GGCGGTTTCCAATCAAGAAAGGGAGGATGATGAGGACACGACTAGCTCGGATATAACCAT

************************************************************

IR40931_A1 GACTATCTTATGTATTAATCAACCAAAGGTGCATATGTTCTATATTATATTTACTTGTTA

IR64_A2 GACTATCTTATGTATTAATCAACCAAAGGTGCATATGTTCTATATTATATTTACTTGTTA

************************************************************

IR40931_A1 TATATTTGAAACAAACGTTGCTACACAATAAGTTTCGTGTGTTTTTTTTAGCAGAGGTAC

IR64_A2 TATATTTGAAACAAACGTTGCTACACAATAAGTTTCGTGTGTTTTTTTTAGCAGAGGTAC

************************************************************

IR40931_A1 TTGCTTGTGCGAAAGAAAAGAGACCGAGCGTAAGGAATGCATGGATGATTGAAGAAATTG

IR64_A2 TTGCTTGTGCGAAAGAAAAGAGACCGAGCGTAAGGAATGCATGGATGATTGAAGAAATTG

************************************************************

IR40931_A1 ATTGGGGACCAAACCAAGACCTTCTCCAAGTTTTCTTTCATCTCACCACGTCACTTTTGG

IR64_A2 ATTGGGGACCAAACCAAGACCTTCTCCAAGTTTTCTTTCATCTCACCACGTCACTTTTGG

************************************************************

IR40931_A1 TCCATGGAAGACAAGAGATAAAGTTTTATACGTTTTGGATTCGGATTCGGGCCTCTTGAT

IR64_A2 TCCATGGAAGACAAGAGATAAAGTTTTATACGTTTTGGATTCGGATTCGGGCCTCTTGAT

************************************************************

IR40931_A1 AGCATCAACTTAAAACGGACCTAGCCGCTGATCTAGAAGGAATTTAGGGGCCCATGAGTA

IR64_A2 AGCATCAACTTAAAACGGACCTAGCCGCTGATCTAGAAGGAATTTAGGGGCCCATGAGTA

************************************************************

**SNP#2**

IR40931_A1 CTTGTTGGAAAGCTTATGGAGTCTACTTTCAGATGGTTTTGGTCCCCAGTCAAAATTCTT

IR64_A2 CTTGTTGGAAAGCTTATGGAGTCTACTTTCAGATGGTTTTGGTCCCTAGTCAAAATTCTT

********************************************** *************

**SNP#3 ABRE**

IR40931_A1 ACCAAACTGCTGGGAATCTGCAAAACAAGCCATGTATCTTATCCAGTCCGAATCTGATTT

IR64_A2 ACCAAACTGCTGGGAATCTGCAAAACAAGCCACGTATCTTATCCAGTCCGAATCTGATTT

******************************** ***************************

**SNP#4 SiteI**

IR40931_A1 GGGTTTTGGGCTTTGTAATTGTGTTGTGGGCTTAGCCCAAGTGGTTGTGCGCACTAGGGC

IR64_A2 GGGTTTTGGGCTTTGTAATTGTGTTGTGGGCTTAGCCCAAGGGGTTGTGCGCACTAGGGC

***************************************** ******************

**Telo box core SNP#5 CARE**

IR40931_A1 AACCCTAGGACGTCCTTAATCATATTTATTCAGTAGCCGTCATCGTTTAGAGTTGGGTTT

IR64_A2 AACCCTAGGACGTCCTTAATCATATTTATTCAGTAGCCGTCATCGTTTAGAGTCGGGTTT

***************************************************** ******

**DRE/CRT**

IR40931_A1 TGCTTAGAATATTCTGTCAAGAACAGTTTTGCCGCTAGATCGGTTTGTGGAACCCAAAAT

IR64_A2 TGCTTAGAATATTCTGTCAAGAACAGTTTTGCCGCTAGATCGGTTTGTGGAACCCAAAAT

************************************************************

IR40931_A1 TAGAGTGCTTAATCATTCATATGCAATTGTGTTGCAATCTATCTTGTTGTTGCTTGTGTT

IR64_A2 TAGAGTGCTTAATCATTCATATGCAATTGTGTTGCAATCTATCTTGTTGTTGCTTGTGTT

************************************************************

**SNP#6 SNP#7**

IR40931_A1 CTTCGATTCACATGCAGGGATTAGCCTTCTCGGCGAGGTCAACCGGGTTTCAGCACGGTT

IR64_A2 CTTCGATTCACATGCAGGGATTAGCCTTCTCGGCAAGGTCAACCGGGTTTCGGCACGGTT

********************************** **************** ********

IR40931_A1 GATAACCGGAGGAGACGTGGTGCTACGATTGCGGGGCTCAGTTGCGTGTTCGTTCAGAAG

IR64_A2 GATAACCGGAGGAGACGTGGTGCTACGATTGCGGGGCTCAGTTGCGTGTTCGTTCAGAAG

************************************************************

IR40931_A1 CCGGATCGAGTTGTGTCGCGACTCCGCCCAAATCGACTATTTATCAATACCTATCGGAAG

IR64_A2 CCGGATCGAGTTGTGTCGCGACTCCGCCCAAATCGACTATTTATCAATACCTATCGGAAG

************************************************************

IR40931_A1 ATCGGGACCTAACATCCCCCATCAAATAGCTATATCATTCTCAAACATTTATAGATGGAT

IR64_A2 ATCGGGACCTAACATCCCCCATCAAATAGCTATATCATTCTCAAACATTTATAGATGGAT

************************************************************

**Telo box core**

IR40931_A1 ATTTAAAACCCTGCTATTTCTGCAACCGTTGCGAGCTAGCTGTCTGAACCTAAAGGCTTA

IR64_A2 ATTTAAAACCCTGCTATTTCTGCAACCGTTGCGAGCTAGCTGTCTGAACCTAAAGGCTTA

************************************************************

**SNP#8**

IR40931_A1 CGAAATGTATCATTTCCACGCTGGGTTATGCAGCCCTATGTGAACTAGGGAATACACCCA

IR64_A2 CGAAATGTATCATTTCCACGCTGGGTCATGCAGCCCTATGTGAACTAGGGAATACACCCA

************************** *********************************

IR40931_A1 TGTGAGAACCAAGCATACCACTAAGGCCATAGGTATAGATGGCCGAATCGGCTGCAAGGC

IR64_A2 TGTGAGAACCAAGCATACCACTAAGGCCATAGGTATAGATGGCCGAATCGGCTGCAAGGC

************************************************************

IR40931_A1 CCGACCTATCGGGCACGACCAAGCCCGGACCTGAGACCAGTCGGGTTACACGACCCAACG

IR64_A2 CCGACCTATCGGGCACGACCAAGCCCGGACCTGAGACCAGTCGGGTTACACGACCCAACG

************************************************************

**SiteII SNP#9 SiteII**

IR40931_A1 TACACGTTGGGCCGGGCTGTGCCCGCCCACATGTTGCACCAACGGCCGAAGCACAATTCA

IR64_A2 TACACGTTGGGCCGGGCTGTGCCCGCCCACATGTTGCACCAACGGCCCAAGCACAATTCA

*********************************************** ************

SiteII SiteII

IR40931_A1 ATAAGACTCGGGCTGTGCCTGGCTGGCCCAAAGGCACGAAAGCCCACCGTGCTCCTCCAT

IR64_A2 ATAAGACTCGGGCTGTGCCTGGCTGGCCCAAAGGCACGAAAGCCCACCGTGCTCCTCCAT

************************************************************

**SNP#10**

IR40931_A1 GGAAAAAAGTCTATTTGCCTCTCGTATCCGGGCCATCTCTTATATGACTGAAATAAGTCT

IR64_A2 GGAAAAAAGTCTATTTGCCTCTTGTATCCGGGCCATCTCTTATATGACTGAAATAAGTCT

********************** *************************************

IR40931_A1 ACTTTGCATTCCTCATCTCTTTTGGTCGTGTCTTATACGGCTGTAATAAGTCTATTTTGG

IR64_A2 ACTTTGCATTCCTCATCTCTTTTGGTCGTGTCTTATACGGCTGTAATAAGTCTATTTTGG

************************************************************

**SNP#11**

IR40931_A1 CTCCCACATCTATTTTGCCTCCATTATTGTTGGAGAGGGCCGCCCCATCGTGCTAAGGCG

IR64_A2 CTCCCACATTTATTTTGCCTCCATTATTGTTGGAGAGGGCCGCCCCATCGTGCTAAGGCG

********* **************************************************

**SiteII SNP#12**

IR40931_A1 GATGCCCAGGCATGGCCCAAAATTCGGACCGAGGCAGCATGAGCCCGATACCAGATAGAC

IR64_A2 GATGCCCAGGCATGGCCCAAAATTCGGACCGAGGCAGCATGACCCCGATACCAGATAGAC

****************************************** *****************

**SNP#13 SiteII**

IR40931_A1 CGAGCCATGTTTGGGTTAGACCAAAACCCCGTGCCATGGGTTGGACCGTTGGGCCCCGGG

IR64_A2 CGAGCCATGTTTGGGTTAGACCAAAACCCCGTGCCATGGGTTGGACCGTCGGGCCCCGGG

************************************************* **********

IR40931_A1 CCTTTTGGCCAACTATAGACATAGGCACTGCAATGTTGCTTGAGAAAAAAAAAATTATAG

IR64_A2 CCTTTTGGCCAACTATAGACATAGGCACTGCAATGTTGCTTGAGAAAAAAAAAATTATAG

************************************************************

IR40931_A1 TCACACTAGATTGGATGTCTATTTGTTAATACTGTTAGGACAAGCACGACTTGTTCCCGC

IR64_A2 TCACACTAGATTGGATGTCTATTTGTTAATACTGTTAGGACAAGCACGACTTGTTCCCGC

************************************************************

**TATA Box**

IR40931_A1 TACATCCCACTCCGATATACAAAGTGTATTTCGACCGCCCCTTGGCCTTCCAGCC**TATAT**

IR64_A2 TACATCCCACTCCGATATACAAAGTGTATTTCGACCGCCCCTTGGCCTTCCAGCC**TATAT**

************************************************************

**TSS**

IR40931_A1 **ATAT**CAACAACTTCTACCCATACTCTCCCATC**TCCAAG**AAGACCATCAGACACTCGGATA

IR64_A2 **ATAT**CAACAACTTCTACCCATACTCTCCCATC**TCCAAG**AAGACCATCAGACACTCGGATA

************************************************************

IR40931_A1 GAGATATATATTCACTGCTCACTAGTAACAAAGCCACAAACTTGGATTTTATTCAAGTGG

IR64_A2 GAGATATATATTCACTGCTCACTAGTAACAAAGCCACAAACTTGGATTTTATTCAAGTGG

************************************************************

**START**

IR40931_A1 ACAACACATACATAATCCCAGCCATCAAGAAAAATCACATTAGCGCGTGGACTATGCGATG

IR64_A2 ACAACACATACATAATCCCAGCCATCAAGAAAAATCACATTAGCGCGTGGACTATGCGATG

*************************************************************

IR40931_A1 TGTGGAGGAGAAGTGATCCCCG

IR64_A2 TGTGGAGGAGAAGTGATCCCCG

**********************

**Supplemental Figure 2**
